# Emotion beliefs: conceptual review and compendium

**DOI:** 10.3389/fpsyg.2023.1271135

**Published:** 2024-01-04

**Authors:** Michael A. Kisley, Josh Shulkin, Margarita V. Meza-Whitlatch, Rhea B. Pedler

**Affiliations:** Department of Psychology, University of Colorado – Colorado Springs, Colorado Springs, CO, United States

**Keywords:** emotion belief, implicit theories, lay theories, emotion mindset, layperson beliefs

## Abstract

Laypeople hold richly divergent beliefs about emotion, and these beliefs are consequential. Specific forms of belief that have been investigated include the usefulness, contagiousness, duration, dependence upon intersubjective experience, cognitively mediated properties, malleability, and hindering properties of emotion, just to name a few. Progress in this emerging sub-field of research has been hampered by the lack of a widely accepted definition of *emotion belief* able to capture all of these dimensions. Correspondingly, there has been a proliferation of different terminologies, constructs, and measures. The present review aims to address these obstacles by defining emotion belief, and subsequently re-considering existing constructs and measures that align with this definition. The latter is presented in the form of a comprehensive compendium of 21 different constructs and associated self-report measures that assess varying components of one’s beliefs about emotions in general and/or about their own emotions, and an additional 5 scales that were designed to measure one’s beliefs about *another’s* emotions. From the more unified conceptualization of emotion belief presented here, critical areas of future research are highlighted.

## 1 Introduction

Despite broad scientific consensus that emotion is relevant to, and serves an important purpose for an individual, a conspicuous lack of agreement appears to exist across laypersons on the role and relevance of emotion to one’s life (Kneeland and Kisley, 2023). Individual differences have been documented in people’s beliefs regarding broad, overarching characterizations of emotions as useful (Chow and Berenbaum, 2012), helpful or hindering (Karnaze and Levine, 2020), and foolish or wise (Netzer et al., 2018). Variation has also been shown across a diversity of more subtle dimensions of emotion belief, such as the extent to which people believe emotions are contagious (Manser et al., 2012), whether happiness is earned or results simply from luck (Joshanloo, 2019), whether emotions are malleable or fixed (Tamir et al., 2007), and whether one’s emotions leave behind a physical residue that can subsequently affect others’ moods (Savani et al., 2011), as just a few examples. It is important to note that such emotion *beliefs*, sometimes described as lay *theories*, *mindsets*, or *concepts*, are to be distinguished from other forms of lay perspectives including but not limited to one’s *preferences* which can be considered to include emotion *attitudes* (*liking* the experience of certain emotions; Harmon-Jones et al., 2011), *judgments* (positive or negative evaluations of one’s own emotional reaction; Willroth et al., 2023) and *values*. The term “values” has been employed in the context of one’s *wanting* to experience certain affective states (Tsai, 2007), and alternatively to refer to what one feels people *should* do with regards to emotional displays and control (Mauss et al., 2010), as examples.

This article aims to promote the scientific investigation of emotion beliefs in several ways, first and foremost by defining what an emotion belief is, and additionally what it is not. Establishing a conceptual grounding for the emotion belief construct, as well as its study and measurement, is critical for this endeavor (see also Becerra et al., 2020). Further, we bring the concept of belief *dimensions* to bear by connecting the concept of emotion beliefs to an existing literature in cognitive science. The second primary purpose here is to comprehensively review existing constructs that satisfy our definition of emotion beliefs in the hopes of providing researchers in this area with a listing of existing concepts and measures (26 in total) that may be of use when designing studies, to illustrate the wide diversity of different emotion beliefs that have been studied, and also to help the field avoid further proliferation of redundant measures. Then we provide a briefer review of constructs and measures that appear to overlap with emotion beliefs but, based on the definitions provided here, do not strictly qualify as beliefs about emotions. This includes *meta*-emotion, *need for affect*, and several other constructs. The purpose of this comparison is to help clarify the convergence and divergence between emotion beliefs and other concepts in the literature. Critical areas for investigation into emotion beliefs are highlighted throughout this review, and further amplified in the final section. But first, a brief review of why emotion beliefs are important.

### 1.1 Why study emotion beliefs?

The study of emotion beliefs has undergone a recent surge in interest and activity (Gonzalez et al., 2020; Kneeland and Kisley, 2023). Among the critical, over-arching questions that are being asked: what leads one to hold specific beliefs about emotion? Unsurprisingly, evidence suggests that one’s early caregivers are likely to be influential [reviewed by Ford and Gross (2019)], as is the broader cultural context in which one develops (Uchida et al., 2009; Savani et al., 2011; Chow and Berenbaum, 2012; Halberstadt et al., 2020). Perhaps of more immediate concern, what is the functional importance of emotion beliefs, if any? Put another way, do laypeople’s beliefs about emotion have actual consequences? Well-replicated findings have highlighted *correlations* between certain beliefs, such as the belief that unpleasant emotions are irrelevant, and specific functional outcomes. These outcomes include, but are not limited to increased experience of negative emotion and symptoms of psychopathology (Ben-Artzi and Mikulincer, 1996; Kneeland et al., 2016), decreased use of cognitive reappraisal and increased use of expressive suppression for emotion regulation (Tamir et al., 2007; De Castella et al., 2013; Veilleux et al., 2015), less desirable social outcomes (Tamir et al., 2007; Karnaze and Levine, 2018), and poorer performance on cognitive reasoning tasks (Karnaze and Levine, 2020). But do emotion beliefs actually *cause* these outcomes?

A growing body of evidence supports the claim that emotion beliefs can lead to consequential functional outcomes for the individual who holds those beliefs (Ford and Gross, 2019). In one study it was shown that controlling for emotional symptoms of psychopathology does not abolish the relationship between emotion beliefs and other functional outcomes, consistent with the interpretation that one’s beliefs about emotion are “not merely byproducts of affective distress” (Veilleux et al., 2021b, p. 8). Longitudinal studies have shown that one’s emotion beliefs can predict future psychological functioning (Tamir et al., 2007; Ford et al., 2018). Of course, experimental manipulation represents a more direct way to demonstrate that emotion beliefs have causative power (Kneeland et al., 2016), and a small number of relevant findings exist. For example, reading a passage that argues for the malleability of emotions leads participants to be more likely to engage in specific regulation strategies during a subsequent emotion induction (Kneeland et al., 2016). Additionally, encouraging participants to adopt a viewpoint that emotions are “helpful” leads to changes in physiological reactivity and the strategies of emotion regulation employed during a distressing film (Karnaze and Levine, 2020). In another study, random assignment to a “rational” emotion beliefs condition lead to reduced experiences of negative emotion during and immediately after viewing a distressing film clip compared to the “irrational” beliefs condition (Predatu et al., 2020b). And finally, dialectical behavior therapy, an empirically supported clinical intervention, specifically targets mistaken emotion beliefs as an important component of treatment for emotional dysfunction and psychopathology (Linehan, 2015).

The beliefs one holds about emotions can also impact other people in important ways (Gonzalez et al., 2020). For example, one’s belief about the controllability of emotion influences the extent to which they are willing to engage in interpersonal support of another individual experiencing negative emotion (Smith et al., 2023). Beliefs held about the emotions of children predict how parents will engage in emotion socialization with their child, including whether they will teach that negative emotions are important or, alternatively, dangerous (Lozada et al., 2016). Such effects are likely to depend upon whether the parent holds *gendered* beliefs, that is emotion beliefs that depend upon the gender of the child in question (Thomassin et al., 2020). Teachers, who are also in a position to influence the emotional development of children, show wide variation in their beliefs regarding the usefulness of the emotion anger, in particular, and this is likely to impact the way they engage with students during emotional situations (Hagan et al., 2020). But the impact of emotion beliefs may also extend well beyond the people an individual knows personally and interacts with regularly. The everyday, commonsensical beliefs that *researchers* hold about emotion – to be distinguished from their scientific, theoretical orientation – influence their decisions about what emotion topics should be studied at all (Gasper et al., 2019). Potentially even more broadly felt are the beliefs about emotion that individuals in positions of power hold. For example, the impetus for certain judicial decisions, which ultimately impact millions of people, can sometimes be traced back to the idiosyncratic beliefs about emotion that U.S. supreme court justices hold, as revealed by their written opinions on cases (Maroney, 2021). In sum, to borrow the phrasing of Ford and Gross (2019), emotion beliefs *matter*, and not just for the individual holding them.

Despite the progress made to date in establishing the importance of emotion beliefs, the field has been hampered by several interconnected obstacles, which the present review aims to address. In lieu of a clear and widely accepted definition of the concept *emotion belief*, a potentially bewildering array of terminologies, theoretical constructs, and measurement tools have emerged (Kneeland and Kisley, 2023). For example, and as considered in more depth below, the following phrases coined by different researchers appear to be pointing toward the same overarching concept, at least according to our analysis: beliefs about emotion, emotion beliefs, emotion mindsets, evaluations of emotion, implicit theories of emotion, lay conceptions of emotion, lay theories of emotion, perceived properties of emotion, theories about emotion, and others. Similar critiques have recently been advanced in areas of research that are adjacent to or overlapping with emotion beliefs. For example, Edwards and Wupperman (2019) argue that study and clinical application of the construct *emotion schemas*, “core beliefs about emotions and emotional experience,” has suffered from “inconsistent operationalization and limited theoretical integration” across different studies and different researchers (p. 3). Juarascio et al. (2020, p. 2) point to “vague or imprecise measurement and terminology” that characterize the investigation of avoidant and intolerant regulation strategies that some individuals employ to deal with their emotions. These authors argue that this has disrupted researchers’ ability to interpret findings and compare them to those from other studies, as well as to a proliferation of psychometric scales that lack internal validity and/or that are highly redundant with other published instruments. They conclude that a review of existing measures represents a productive and important way to uncover the common, underlying core construct of interest. Conceptualization of the present article is indebted to these critiques, though the focus here is on *emotion beliefs* specifically, which is defined in more depth below. This construct will also be carefully distinguished here from adjacent and overlapping constructs including but not limited to emotion schemas and avoidant/intolerant regulation strategies mentioned above. We intend this approach to help address the need to better “conceptually organize” emotion beliefs in order to promote the development of theory and subsequent empirical research (Gonzalez et al., 2020).

### 1.2 Belief

Careful consideration of the terminology employed in the emotion beliefs literature reveals that researchers tend to confound the concept of “believing” something is true with “thinking,” “feeling,” or “agreeing” that something is true. This became apparent upon our review of the language commonly employed in psychometric scales designed to assess emotion beliefs as we were compiling the compendium included below. For example, consider the Evaluations of Emotions scale, which aims to assess “how people think” about specific emotions, but which nevertheless “reflects beliefs” that they hold (Netzer et al., 2018). The instructions for this scale ask participants to answer items in a way that “best describes what you think,” and the Likert scale is subsequently explained to represent “your feelings” about the statements.^1^ The Emotion and Regulation Beliefs Scales (Veilleux et al., 2015) instructs participants to “select the degree to which you agree with each statement.” By contrast, the Individual Beliefs About Emotions Scale (Veilleux et al., 2021a) asks participants to “select the option closest to your beliefs about emotions.” Are these scales, with their diverse phrasing, all measuring the same thing? And if so, is that thing “belief?” Closer examination of the concept of *belief* may help answer these questions.

Recently, psychological scientists have shown increased interest in defining and understanding belief [reviewed by Camina et al. (2021)]. The most common approach has been to build from the foundation offered by philosophers, and as explained by Schwitzgebel (2019): belief is a “propositional attitude,” where a proposition is a statement (e.g., the sun will rise tomorrow), and an attitude is a particular mental state, or “stance,” that includes but is not limited to hope, desire, imagining, and belief.^2^ What distinguishes a belief from these other states is that a belief can be falsified, though of course one’s intention is typically to hold beliefs that are true. Put another way, beliefs “ought to fit with, or get it right about, or match up to” reality (Section 1.1.2). Camina et al. (2021) argue that this definition does not disambiguate belief from other mental states including, for example, opinion and knowledge, as these can also end up being falsified in light of new evidence. Their approach to uniquely defining belief is as follows: if an individual answers “no” to the hypothetical question “If you were given irrefutable evidence against it, would you change your mind?” then the proposition in question is a belief. We find this definition restrictive, and more likely to represent a sub-class of belief, perhaps *unwavering belief*. Regardless, the definition of belief employed for the present review ought not be considered as universally agreed-upon, and in some instances may overlap with other constructs, particularly *opinion* and *knowledge*. In such cases, we suggest that these terms be taken to reflect varying levels of certitude of belief rather than qualitatively different mental states, at least as a starting point.

In order to provide a conceptual framework to guide scientific research on belief Connors and Halligan (2015) developed a cognitive account from which to work. They conclude that beliefs provide “the basis for us to understand the world and act within it” (p. 2). Their account, which builds from the philosophical foundation described above, posits several important *functions* of beliefs, including a representational and explanatory framework of the world and one’s place within it. As such, beliefs allow one’s behavior to reflect broader, more comprehensive notions of the world, rather than simply being reflexive responses to whatever immediate sensory inputs are currently present. Believing that the sun will rise tomorrow allows one to plan ahead, rather than simply reacting to the presence of the sun when it appears. From a biopsychological perspective, belief provides “a means for increasing the efficiency of brain mechanisms involved in” solving problems, making decisions, setting goals, and moving through the world (Seitz and Angel, 2020, p. 5).

Connors and Halligan (2015) delineate several pragmatic *dimensions* of belief, only some of which will be considered here. For example, beliefs develop from different sources, such as from one’s own experience, and/or the influence of a trusted source: in my experience the sun has arisen every day; also, professional forecasters and scientists reassure me this will continue to happen. Beliefs differ in their availability to awareness, from implicit (affecting one’s behavior without conscious access) to more “reflective.” Scope varies too, as some beliefs refer to individual cases (e.g., this fruit I found will provide nourishment), others to groups (the fruits in this forest will provide nourishment), and others to whole classes (all fruits provide nourishment). Further, a belief’s degree of personal reference can vary, for example distinguishing between the belief “my thoughts are useful to me” from the belief “thoughts are useful.” All these dimensions of belief will be examined closely in the next two sections of this review as they relate to emotion. Additional dimensions put forward by Connors and Halligan (2015) will be considered in the final section of this review, where we consider important future directions for research in the study of emotion beliefs.

### 1.3 Emotion belief

Here we define a reflective emotion belief as a *statement about emotion that an individual endorses as true, or likely to be true*. For example, “emotion helps people focus on what’s important” (Karnaze and Levine, 2020, p. 19). Whether a layperson states that they think, feel, agree, believe, or otherwise endorse that this is true, it seems reasonable to consider it an emotion belief. That being said, the level of conviction expressed may vary across these terms in common usage.^3^ For example, instead of believing a statement *is true*, one who states they “think” something is true may be signaling their belief that the statement *is likely to be true*. In order to avoid prematurely narrowing subsequent research directions on emotion beliefs, this definition was intentionally devised to avoid explicit reference to the specific sources and functions of beliefs reviewed above. Nevertheless, and adapting the ideas of Connors and Halligan (2015), emotion beliefs likely reflect the accumulation of one’s experiences with emotion, as well as the influence of outside sources including but not limited to caretakers and broader culture. Consequently, these beliefs provide one with guidance for appropriately enacting (or not enacting) emotions into behavior, as well as a way to understand emotions and emotional behaviors in oneself and in others.

Defining *emotion* in this context is more challenging. Various theoretical frameworks (evolutionary, appraisal, constructionist, interoceptive inference, etc.) generally posit different definitions of emotion. However, it is not researchers’ definition of emotion that is relevant here, rather the understanding of “emotion” that laypeople hold, and how that intersects with any instructive language that researchers use when engaging participants. For example, the Emotion Beliefs Questionnaire (Becerra et al., 2020) instructions provide participants with examples of discrete emotions (sadness, fear, happiness, joy, etc.) to help them understand what is meant by the word “emotion.” This is quite typical of emotion belief measurement approaches, although there are important variations as described below in the compendium of measures. This approach is also consistent with common-use dictionary definitions for which emotions are generally characterized as “feelings,” often contrasted with thoughts, and examples are provided, most commonly love, anger, joy, hate, and fear.^4^

It can be instructive to examine how the definition of emotion belief presented here relates to the multidimensional nature of beliefs generally, and as elaborated by Connors and Halligan (2015). The primary focus here is on explicit emotion beliefs upon which one can reflect and voluntarily report, in contrast to implicit emotion beliefs that may nevertheless impact one’s behavior involuntarily. Although the latter is certainly of interest, as evident from the review of measures below, there is a paucity of methods for assessing implicit beliefs. The vast majority of emotion belief assessments require self-report which depends upon one’s ability to consciously *reflect* on their beliefs. Considering another dimension of belief, the general statement “emotion helps people focus on what’s important” can be compared to a statement of more limited scope, such as “*fear* helps people focus on what’s important” (individual case of a discrete emotion), or “*negative emotion* helps people…” (group of emotions). Regarding personal reference, the general statement above can be contrasted with a more personal one: “*my* emotion helps *me* focus on what’s important” (e.g., De Castella et al., 2013). Additional dimensions of emotion belief have been described including but not limited to those related to different emotion intensities and different situational contexts (Ford and Gross, 2019).

The focus of the present review is beliefs that posit emotion (or emotions) as the primary object of belief, such as the statement “anger is useful.” This is to be contrasted to beliefs about one’s emotional experiences, strategies, abilities, or preferences (Veilleux et al., 2015, 2021a). Take for example the statement “when I am angry, the feeling overwhelms me.” Technically, this satisfies the definition of an emotion belief, as it is a statement to which an individual can signal their endorsement, and emotion is an important part of the statement. However, this primarily relates to an individual’s *experience* of emotion, as opposed to what they believe *about* emotion. This characterization applies to constructs such as anxiety sensitivity (Taylor et al., 2007) and distress tolerance (Simons and Gaher, 2005). Statements about emotional *strategies* and *behaviors* that are common in the emotion regulation literature (Ford and Gross, 2019; Juarascio et al., 2020), such as the use of cognitive reappraisal, expressive suppression, or avoidance, would also not be considered emotion beliefs by the definition advanced here. It is also important to distinguish statements regarding one’s beliefs about their own *self-efficacy* to successfully regulate emotion from their beliefs about emotions *per se* (Becerra et al., 2020). E*motional intelligence* would not be considered a form of emotion beliefs, because the primary object of belief is an individual’s *abilities* or *skills* to recognize, understand, and appropriately express emotions (Mayer et al., 2008; Gómez-Leal et al., 2018). Other constructs that focus primarily on one’s emotional skills and abilities include but are not limited to *alexithymia* (Bagby et al., 1994) and *attention to emotion* (Boden and Thompson, 2017). Finally, also to be contrasted to emotion beliefs is preferential *attitudes* such as “I like being scared” (Harmon-Jones et al., 2011), *judgments* about one’s emotions (e.g., “I almost always consider my positive emotions appropriate,” Willroth et al., 2023) and *valuations* that include how one would “IDEALLY like to feel” (Tsai et al., 2006).

## 2 Compendium of emotion belief constructs and measures

In this section we compile and describe existing constructs and associated measures that satisfy the definition of emotion beliefs provided above, despite apparent differences in terminology. The focus here is on laypeople’s beliefs *about emotion*, as opposed to beliefs about an individual’s emotional attributes, experiences, and behaviors. The measures described here depend on self-report and should therefore be understood as assessing *reflective* beliefs. In addition to describing relevant concepts and assessment, for each entry, we highlight some of the belief dimensions delineated above: whether the emotion belief is personal (about *my* emotions) or general (about emotions generally); and whether the belief refers to specific discrete emotions (e.g., fear, anger, happiness) or class of emotions (positive or negative emotions) or all emotions more generally. Cutting across these dimensions, many beliefs reflect broad statements concerning the extent to which emotion is useful (i.e., of utility), be it all emotion, my emotion, discrete emotions, etc. In more colloquial language, does an individual believe emotions are “good or bad” (Ford and Gross, 2019, p. 74)? “Friendly or unfriendly” (Veilleux et al., 2021b, p. 2)? “Helpful or hindering” (Karnaze and Levine, 2020)? “Foolish or wise” (Netzer et al., 2018)? The second of two so-called “superordinate” emotion beliefs relates to the extent to which emotions are believed to be malleable or controllable (Ford and Gross, 2019; Becerra et al., 2020). Other beliefs described here include those related to the causes of emotions, how long emotions last, whether they can be contagious, whether they are determined by genetics or by circumstances, etc. Table 1 provides a brief summary of these belief dimensions for the constructs and scales reviewed in this section.

**Table 1:** Emotion belief constructs and scales that refer to emotion in general or to one’s own emotion.

Construct/Scale	Belief dimensions							
	Type			Scope		Target		
	Utility	Malleability	Other	Personal	General	All	Pos/Neg	Discrete
Beliefs about emotional residue (Savani et al., 2011)			**✓**		**✓**	**✓**		
Beliefs about emotions (Manser et al., 2012)	**✓**		**✓**	**✓**			**✓**	
Beliefs about grief-related emotions (Zhou et al., 2023)	**✓**	**✓**			**✓**			**✓**
Cognitive mediation beliefs (Turner et al., 2021)		**✓**	**✓**	**✓**			**✓**	
Emotion and regulation beliefs (Veilleux et al., 2015)		**✓**	**✓**		**✓**	**✓**		
Emotion beliefs (Becerra et al., 2020)	**✓**	**✓**			**✓**		**✓**	
Emotion mindset (Skymba et al., 2022)		**✓**			**✓**	**✓**		
Essentialist beliefs about happiness (Choi et al., 2021)		**✓**	**✓**		**✓**			**✓**
Evaluations of emotions (Netzer et al., 2018)	**✓**				**✓**			**✓**
Help and hinder theories about emotion (Karnaze and Levine, 2020)	**✓**				**✓**	**✓**		
Implicit theories of emotion (Tamir et al., 2007)		**✓**			**✓**	**✓**		
Implicit theories about one’s own emotions (De Castella et al., 2013)		**✓**		**✓**		**✓**		
Individual beliefs about emotion (Veilleux et al., 2021a)	**✓**	**✓**	**✓**	**✓**	**✓**	**✓**		
Lay conceptions of happiness (Joshanloo, 2019)		**✓**	**✓**	**✓**	**✓**			**✓**
Lay theories of emotion (Ben-Artzi and Mikulincer, 1996)	**✓**	**✓**	**✓**		**✓**	**✓**		
Lay theories of emotion transience (Labroo and Mukhopadhyay, 2009)			**✓**		**✓**	**✓**		
Negative emotion appraisals (Babij et al., 2020)	**✓**			**✓**			**✓**	
Perceived utility of emotion (Chow and Berenbaum, 2012)	**✓**			**✓**			**✓**	
Shame-related beliefs (Li et al., 2023)	**✓**			**✓**				**✓**
Stress mindset (Crum et al., 2013)	**✓**			**✓**				**✓**
Theories of anxiety (Schroder et al., 2015)		**✓**			**✓**			**✓**

“Type” refers to the form of emotion belief targeted: “Utility” corresponds to beliefs related to the usefulness of emotion; “Malleability” refers to beliefs regarding the potential to change emotions, or their controllability; “Other” corresponds to other types of emotion beliefs. “Scope” concerns the referential aspect of the belief: whereas “Personal” refers to beliefs about one’s own emotions, “General” corresponds to beliefs about emotion more broadly.” Target” corresponds to the type of emotion targeted by the scale: “All” means that the belief applies to all emotion (see Section 2.1), “Pos/Neg” indicates that the scale targets either positive or negative emotion, or both but measured in a separable manner (Section 2.1), and “Discrete” implies that beliefs are measured for some number of specific, discrete emotions (happiness, grief, anger, sadness, etc.; Section 2.2). For more details about each construct/scale, see 2. Compendium of Emotion Beliefs Constructs and Measures.

This compendium is intended to provide a listing of lay emotion belief concepts and measures, each of which may be of use for researchers in this area to consider. Inclusion here does not imply endorsement or criticism. We leave it to each reader to assess rigor and validity based on their own review of the cited articles. Additionally, one of the goals of this effort is to help integrate social and clinical psychology literatures on emotion beliefs. That being said, we only describe constructs and measures that are intended for research. So, for example, we do not discuss *emotion myths*, which would satisfy the definition of emotion beliefs provided here, but is used only as a clinical tool for helping dispel “dysfunctional beliefs” about emotions in dialectical behavior therapy (Linehan, 2015). Additionally, for those who do not find the “perfect” scale for their research in this listing, we encourage consideration of scale adaptation using one of the following instruments as a starting point. Finally, despite our best efforts, we may have missed some relevant scales, and undoubtedly more will be developed after the publication of this article. Please reach out to the corresponding author to share candidate scales and to receive an updated listing.

Section 2.1 describes emotion belief concepts and scales that relate to emotion very broadly, or to a class of emotions (e.g., positive emotions), or combine beliefs about a number of discrete emotions into a summary score. Section 2.2 describes constructs and measures that characterize instead beliefs about a smaller subclass of emotions (e.g., grief-related emotions) or a single discrete emotion (e.g., happiness).

### 2.1 Beliefs about emotions in general and about one’s own emotions

#### 2.1.1 Beliefs about emotional residue

Laypeople vary in the extent to which they believe emotions “emanate” from an individual and leave a physical “residue” behind in a space that can affect the emotions and behaviors of others who enter that space (i.e., a shared physical area such as a room). Savani et al. (2011) anchored their investigation of this particular emotion property on the *law of contagion*, which concerns the “perceived transfer of some essence or property from the contaminated object to the uncontaminated one” (p. 684). They employed a brief Likert self-report scale to measure beliefs about emotional residue that distinguishes between within-body and between-body transmission of emotions in general (i.e., not specific emotions). An example item from the latter form of transmission: “Do you believe that emotions can leave the human body and enter the outside world in the form of a physical substance?” (p. 687). Cultural differences between Americans and Indians were demonstrated for this measure. Next the authors described scenarios involving emotional situations, and asked participants the extent to which they “believe” the emotion of one individual would impact another who enters a space inhabited previously by the former. Each scenario involved a different discrete emotion, such as happiness or sadness. As such, and taken as a whole, the effects found across measures suggest that beliefs about emotional residue appear to apply to emotion broadly, but also to particular instances of emotions. Importantly, Savani et al. (2011) also devised a scenario-based approach to assess *implicit* beliefs by avoiding asking about emotion transmission explicitly. They found that many people who do not endorse an explicit belief in emotional residue nevertheless responded in a way that is consistent with the maintenance of an implicit belief. This discrepancy highlights the need for further development of means for assessing implicit beliefs throughout the broader emotion beliefs field of study.

#### 2.1.2 Beliefs about emotions

The Beliefs About Emotions Questionnaire was developed to assess beliefs about emotions as a “metacognitive construct,” following from existing scales that measure beliefs about cognition (Manser et al., 2012). The authors were specifically concerned with beliefs that emotions are “invalid, inaccurate, shameful” (p. 236), and how this may impact emotion regulation as well as potentially contribute to the maintenance of psychopathology and distress. In addition to drawing inspiration for items from a previously published metacognitive questionnaire, the authors considered mentalization, dialectical behavior therapy, and emotion focused therapy literatures. All items use the phrase “feeling upset” or some variation thereof, instead of the word emotion or specific discrete emotions.^5^ The vast majority of items employ personal pronouns (I, me, my), with a few exceptions that could be considered to reflect more general beliefs, such as “It is silly to feel upset” (p. 240). The final items were found to load on the following 6 factors: beliefs that emotions are overwhelming and uncontrollable, shameful and irrational, invalid and meaningless, useless, damaging, and contagious. Based on item wordings, all these factors except the first represent beliefs *about* emotion. By contrast, most of the items in the overwhelming and uncontrollable factor appear to represent beliefs about one’s individual emotional *experiences*, for example “When I’m upset, that feeling takes over completely.” The authors found that participant responses predicted variance in the reporting of anxiety, depression, and borderline personality disorder symptoms.

#### 2.1.3 Cognitive mediation beliefs

Beliefs about what can cause and what can change an emotional response are likely to influence the extent to which one engages in antecedent-focused regulation strategies such as cognitive reappraisal (Turner et al., 2021). It is worth noting that these dimensions of beliefs are subtly different from the idea that emotions are controllable more generally. The Cognitive Mediation Beliefs Questionnaire allows for the characterization of beliefs along 2 factors: emotions are responses to external stimuli or events (the stimulus–response generation viewpoint), and emotions, being “cognitively mediated,” arising from cognitive appraisals of stimuli or events, can therefore be changed through cognitive strategies (the cognitive-mediation change viewpoint). Scale items refer to one’s own emotions, or more often “feelings.” Scale instructions ask participants to rate the extent to which they agree with (“I believe that…”) statements about “unpleasant or unwanted emotion.” Example items: “My emotions are caused entirely by others’ actions toward me,” from the stimulus–response generation viewpoint, and “To change how I feel, I can change my thoughts about the situation,” from the cognitive-mediation change viewpoint (p. 941). Of note, correlation between factor scores was only moderately negative, consistent with the idea that one could potentially hold beliefs consistent with both viewpoints. As predicated, higher scores on the cognitive-mediation change factor and lower scores on the stimulus–response generation factor predicted more common attempts to engage in cognitive reappraisal, as well as greater well-being and lower emotional reactivity.

#### 2.1.4 Emotion and regulation beliefs

The authors of the Emotion and Regulation Beliefs Scale aimed to develop an instrument that would measure people’s beliefs that emotions can “hijack” self-control, that attempting to regulate one’s emotions is worthwhile, and that emotions can “constrain” behavior (Veilleux et al., 2015). For their initial pool of items, they drew from a broad range of research and clinical literatures including but not limited to implicit theories of emotion (reviewed below; Tamir et al., 2007) and beliefs about emotional contagion (reviewed above; Savani et al., 2011). Items were worded with a general, as opposed to personal, framing, such as “Emotions make people lose control” (p. 91). The intended scope of belief appears to be primarily negative emotion, although this is not explicitly stated in the instructions to participants, or within most of the items, which just use the word emotion(s). A small minority of items are more specific, for example including “angry,” “feeling down,” or “sadness.” The underlying factors aligned with the original intention of the authors, and were labeled emotion constraint, regulation worth, and hijack. Subsequent analyses of divergent validity provide evidence that these classes of beliefs about emotion are distinct and distinguishable from beliefs about one’s own emotional experiences and perceived regulation self-efficacy.

#### 2.1.5 Emotion beliefs

Conceptualized as a tool for assessing beliefs about emotion controllability and usefulness, both considered to be “superordinate” beliefs following Ford and Gross (2019), the Emotion Beliefs Questionnaire was also designed to separate beliefs about positive and negative classes of emotions (Becerra et al., 2020). Item development followed from a review of existing emotion belief instruments including implicit theories of emotion (reviewed below; Tamir et al., 2007) and the emotion and regulation beliefs scale (reviewed above; Veilleux et al., 2015). Additional sources for inspiration were other emotion-related constructs including attitudes toward emotion (Harmon-Jones et al., 2011) and parents’ beliefs about children’s emotions (Halberstadt et al., 2013). Scale items were worded to assess beliefs about emotion in general (as opposed to one’s own emotion). Examples of discrete emotions were provided in the instructions to help participants understand what was meant by the item-level phrases “negative emotion” (“e.g., sadness, fear, and anger”) and “positive emotion” (“e.g., happiness, joy, and amusement”). An example controllability item is “Once people are experiencing positive emotions, there is nothing they can do about modifying them” (p. 11). Several models were tested, the strongest consisting of an overall controllability factor that included positive and negative emotion items, and separate usefulness factors for positive and negative emotion items. Interestingly, belief in the usefulness of negative emotion was found to be more strongly endorsed overall than belief in usefulness of positive emotion.

#### 2.1.6 Emotion mindset

The Emotion Mindset Scale (EMS) scale borrows items from other instruments targeting malleability beliefs, including the Implicit Theories of Emotion Scale (described below; Tamir et al., 2007), as well as some novel items (Skymba et al., 2022). Although all items appear to satisfy the definition of emotion beliefs advanced here, the single-factor scale combines individual items that refer either to the emotions of “people” or, alternatively, “teens,” thus potentially confounding the target of belief. Nevertheless, the authors explain that they selected wording to emphasize general as opposed to personal beliefs. Within samples of adolescents, greater malleability beliefs on this scale were associated with less emotion dysregulation and fewer symptoms of depression.

#### 2.1.7 Help and hinder theories about emotion

Laypeople hold beliefs about how helpful or hindering emotions are to daily function, but these two dimensions are not necessarily opposite ends of a single spectrum. As such, the Help and Hinder Theories about Emotion Measure was developed to measure these dimensions separately (Karnaze and Levine, 2020). Items, drawn from functionalist theories of emotion and other literatures, were designed to assess people’s beliefs about emotion “overall,” without reference to their beliefs concerning controllability or emotion regulation. They specifically aimed to measure “global” beliefs about emotion as opposed to specific discrete emotions. Further, the instructions specify that participants should consider both positive and negative emotions when answering items. An example item from the help factor is “Emotion is a strength that humans have” (p. 19). The word “weakness” is used for the corresponding item in the hinder factor. Participants that scored higher on help theory beliefs endorsed greater well-being and more common use of cognitive reappraisal as an emotion regulation strategy, whereas those scoring higher on hinder theory endorsed lower well-being and more common use of expressive suppression. But help and hinder factor scores were not significantly correlated, supporting the claim that one’s belief about the helpfulness and hindrance of emotion are not mutually exclusive (see also *lay theories of emotion* below; Ben-Artzi and Mikulincer, 1996).

#### 2.1.8 Implicit theories of emotion

An implicit theory, also sometimes referred to as implicit belief, lay- or folk-theory, and mindset, represents the assumptions inherent to one’s worldview, providing meaning and a basis for that individual’s experience of reality (Dweck et al., 1995). The Implicit Theories of Intelligence scale was developed to contrast entity beliefs, that intelligence is relatively fixed, and incremental beliefs, that intelligence is malleable (Dweck, 1999). This served as the model for the Implicit Theories of Emotion scale (ITES; Tamir et al., 2007): emotion would be characterized as malleable and controllable by incremental theorists, but fixed and uncontrollable by entity theories. It is important to note that despite aiming to characterize “implicit” beliefs, this scale depends upon self-report. Scale wording targets general beliefs about the malleability and controllability of emotion, as opposed to one’s personal belief about their own emotions, and emotion broadly as opposed to specific discrete emotions. An example item that is consistent with entity beliefs: “No matter how hard they try, people cannot really change the emotions they have” (p. 735). Scores on the single scale factor, entity vs. incremental beliefs, were found to predict the use of cognitive reappraisal to regulate emotions, as well as social and emotional adjustments during a major life transition. Participants endorsing entity beliefs reported lower well-being, more symptoms of depression, and less social support during their first year of college. Interestingly, people who held entity beliefs about emotion in general also tended to believe that their own emotion regulation self-efficacy was lower, suggesting a possible link between these different types and scopes of belief.

The ITES has been adapted in several ways, including a measure of one’s beliefs about malleability of *their own* emotions (next entry; De Castella et al., 2013), as well as beliefs about the malleability of *another’s* emotions (described in the next section; Smith et al., 2023). Versions have also been developed with slightly altered language for a younger sample (14–18 years of age; Ford et al., 2018), phrased in the second person (e.g., “If you want to, you can change the emotions you have,” p. 179; King and Dela Rosa, 2019), and targeting anxiety instead of emotion more broadly (Theories of anxiety, described below; Schroder et al., 2015).

#### 2.1.9 Implicit theories (beliefs) about one’s own emotions

Beliefs about emotion in general, such as those assessed with the Implicit Theories of Emotion Scale (immediately above; Tamir et al., 2007) may not always align with beliefs about one’s own emotions (De Castella et al., 2013). To assess the extent to which such beliefs may differ on the property of malleability, these authors modified all items of the Implicit Theories of Emotion scale in order to measure “personal beliefs about the malleability of emotions” (p. 499). As an example, the scale item described immediately above became “No matter how hard I try, I cannot really change the emotions that I have” (p. 499). It was found that average entity beliefs ratings (i.e., emotions are *not* malleable) were significantly lower for personal beliefs compared to general beliefs. This finding suggests that people tend to view their own emotions as more malleable than they consider emotions in general to be. More broadly, it confirms that one may hold discrepant beliefs about their own emotions and emotions in general. Personal emotion beliefs were found to be stronger predictors of well-being and psychological distress than general emotion beliefs. This provides additional evidence for the importance of careful examination of, and delineation between different dimensions of emotion belief.

#### 2.1.10 Individual beliefs about emotion

The Individual Beliefs About Emotion scale (IBAE) was developed to quickly assess several different dimensions of emotion belief, and to be appropriate for clinical and research settings (Veilleux et al., 2021a). The conceptual starting point for this instrument was Leahy’s emotional schemas scale (Leahy, 2002), but with an increased focus on one’s beliefs *about emotion*, and further without including questions regarding one’s emotional symptoms, behaviors and experiences. Nine different dimensions of belief are assessed by single questions, each of which includes unique anchor terms. For example, belief about the cause of emotions is assessed with the question “Where do emotions come from?” The left-most Likert anchor states “Emotions happen because of clear identifiable causes,” and the right-most anchor reads “Emotions come from out of the blue, for no reason” (p. 1075). The other eight dimensions of emotion belief assessed in this way are judgment regarding negative emotions (are they useful or destructive), complexity, expression (should they be shared with others), preference (thought or feeling; technically this item is not an emotion belief as defined in the present review), behavior control (do emotions control behavior), malleability, uniqueness (are your emotions different from others’?), and longevity. Scale instructions guide participants to respond based on their “own beliefs about emotions.” No discrete emotions or emotion classes are mentioned except for the judgment item. Based on the wording of questions and anchors, some items reflect beliefs about emotions in general, whereas others reflect beliefs about one’s own emotions. The final item is a dichotomous yes/no question: “Do your beliefs about emotions (all of the above) *change* when you are in a strong emotion?” (p. 1074). Even though the IBAE does not include items related to one’s emotional *symptoms*, emotion beliefs reported through several scale items predicted symptoms of psychopathology.

#### 2.1.11 Lay theories of emotion

This construct and scale arose from the observation that laypeople, “in their attempt to understand and control their inner and outer worlds, generate hypotheses and develop theories about ‘what things are’” (Ben-Artzi and Mikulincer, 1996, p. 249). This is similar to the concept of implicit theories described above (Dweck et al., 1995). Such lay theories are argued to allow for the development of goals and actions. Candidate scale items were drawn from open-ended, “non-reactive” responses to cues to report on the “attributes” that people associate with emotions. However, the final version of the scale utilizes wording that asks participants to rate their level of agreement with statements that reflect emotion beliefs, for example the items “Emotions are illogical,” from the bizarreness factor, and “Emotions are powerful,” from the intensity factor (p. 258). The other factors reflect beliefs about the experiential significance, potential for disturbance, instability, potential for cognitive interference, motivational power, and controllability of emotions. It could be argued that participant responses to these items may be more reflective of their beliefs about their own *experiences* of emotion, rather than beliefs *about emotion*. But, at least, that is not how the scale is worded. Two higher-order factors emerged: the belief that emotions pose “threat,” and that emotions provide “benefit.” These two factors were found to be orthogonal (i.e., not correlated), suggesting that one can simultaneously hold both classes of emotion belief (see also *help and hinder theories of emotion* above, Karnaze and Levine, 2020).

#### 2.1.12 Lay theories of emotion transience

People’s beliefs regarding the amount of time an emotion will last is often quite different from the actual duration of an emotional reaction, and these beliefs are relevant to one’s “search for happiness” (Labroo and Mukhopadhyay, 2009). For example, a happy person who believes that emotions are *fleeting* may take immediate measures, such as indulging in pleasurable foods or activities, in an attempt to sustain that emotion. An unhappy person who believes that emotions are *lasting* would be predicted by these authors to engage in regulation as well, but in this case to change their emotional state. Several different approaches across multiple studies were employed to assess beliefs about emotion transience. After finding supporting evidence for implicit transience beliefs through indirect and subtle experimental manipulations, a study was conducted in which participants were asked directly about their beliefs. For this the authors created a Perceptual Inclinations Inventory which was adapted from Tamir et al.’s (2007) implicit theory scale (see above) to focus on beliefs about emotion transience. Wording of items included the word “emotions,” as opposed to specific discrete emotions, and participants were asked about emotion in general as opposed to their own emotions. For example, “In general, emotions that people experience are…,” to which a participant makes a Likert rating between 1 = “short-lived, fleeting, tend to fade in a short while” and 7 = “persistent, lasting, endure for a long while” (p. 250). As predicted, individual differences in beliefs about emotion transience predicted people’s attempts to regulate their emotions, depending on their current emotional state.

#### 2.1.13 Negative emotion appraisals

When encountering roadblocks to achieving personal goals, people tend to appraise negative emotions experienced at these times as either “enhancing” or “debilitating” their ability to overcome the setback (Babij et al., 2020). This framework was directly adopted from that of *stress mindset* (Crum et al., 2013), which is also described below in the compendium. Essentially, items for the Negative Emotions Appraisals measure were modified by replacing the word “stress” with the phrase “negative emotion.” An example item is “Experiencing negative emotion enhances my ability to reach my goal” (p. 448). Because 6 of 8 items refer to one’s self, we consider this scale to primarily reflect personal, as opposed to general, beliefs about emotion. The authors found that individuals holding a negative “emotion-is-enhancing” appraisal tended to experience less severe negative emotion in response to a setback, but this result did not replicate in a second study.

#### 2.1.14 Perceived utility of emotion

There are individual and cultural differences in the perceived utility of discrete emotions, which is defined as “the representation of the usefulness of specific positive and negative emotions in goal attainment” (Chow and Berenbaum, 2012, p. 55). Although the word “perceived” is used here, the items of the Perceived Affect Utility Scale (PAUSe) are worded in a manner that is consistent with *beliefs* about the usefulness of emotions. For example, “Feeling *happy* lets me know that I am living up to my expectations,” and “When I fail to meet my expectations in something, feeling *embarrassment* motivates me to do better in achieving my goals and expectations the next time around” (p. 57). All scale items focus on beliefs about one’s own emotions (as opposed to emotions in general), and each item mentions either a positive self-centered emotion (happy, proud, deserving), a positive other-centered emotion (appreciation, humility, respectful), a negative self-centered emotion (anger, jealousy, disgust), or a negative other-centered emotion (embarrassment, guilt, shame). As predicted by the authors, endorsement of beliefs in the usefulness of self-centered emotions was associated with self-construed independence, and beliefs in the usefulness of other-centered emotions was associated with self-construed interdependence, as well as dutifulness and self-discipline. These findings suggest the existence of culturally relevant discrepancies in emotion beliefs concerning the usefulness of different emotions, especially when comparing self-centered and other-centered emotions.

### 2.2 Beliefs about specific emotions

#### 2.2.1 Beliefs about grief-related emotions

Two scales were developed to investigate beliefs about the “goodness,” or usefulness (e.g., “Grief-related feelings such as sorrow, anger, or worries help people face their loss directly”), and the controllability (e.g., “If they want to, people can change the grief-related feelings that they have,” p. 5) of grief-related emotions (Zhou et al., 2023). The latter was adapted from Tamir et al.’s (2007) Implicit Theories of Emotion Scale, described below. All items, with one exception, are stated with regard to grief-related emotions in general, as opposed to one’s own grief-related emotions. Beliefs about grief-related emotions were found to mediate the relationship between social acknowledgement of a loss, and the prolongation of grief symptoms, and this finding crossed both cultures studied: bereaved individuals from China and Switzerland.

#### 2.2.2 Essentialist beliefs about happiness

Essentialism refers broadly to a belief in essential, immutable characteristics that are naturally present within members of a group, often due to biological inheritance (e.g., genetic influences). The authors of the Essentialist Beliefs about Happiness (EBH) scale were interested in the extent to which people believe that happiness, specifically, is essential in this manner (Choi et al., 2021). Items were selected and confirmed to load on to 3 factors: the biological basis component which corresponds specifically to genetic determinism of happiness (e.g., “Happiness is genetically determined”), the effort constructivism component which relates to the potential for one to change their level of happiness through effort and practice (e.g., “Even unhappy people can attain happiness if they strive to become happy”), and the immutability component which reflects a more unchanging nature of happiness (e.g., “In general, a person’s happiness level does not change much throughout one’s lifetime,” p. 440). All items are worded in a general manner, referring to “one” or “people,” as opposed to referring to “me” or “my” happiness. As predicted, those who endorsed more essentialist beliefs about happiness were less likely to undertake activities that typically boost one’s happiness.

#### 2.2.3 Evaluations of emotions

Attitudes toward emotion are considered to be characterized by an evaluative assessment, such as liking or preferring the experience of an emotion (Harmon-Jones et al., 2011). The authors of the Evaluations of Emotions scale (EVE) delineate three separable components to such attitudes: affective, behavioral, and cognitive (Netzer et al., 2018). They argue that the Attitudes Toward Emotions scale (ATE; Harmon-Jones et al., 2011) measures the first two of these, but does not capture the cognitive component, which “is related to how people think about the attitude object…[and] reflects beliefs about the object” (p. 14), where the object is one of a number of discrete emotions (fear, disgust, happiness, anger, or sadness). As such the cognitive component, but not the other components, satisfies the definition of emotion belief advanced here. For each discrete emotion factor, participants were asked to rate the extent to which they “think” each emotion is worthless vs. valuable, foolish vs. wise, redundant vs. necessary, harmful vs. useful, bad vs. good. Items were worded generally, as opposed to personally. Netzer et al. found that the EVE was distinct from the ATE, and was also generally more strongly correlated with the perceived utility ratings of emotions, whereas the ATE was more strongly correlated with the perceived pleasantness ratings of emotions, as predicted.

#### 2.2.4 Lay conceptions of happiness

Across cultures, people tend to hold very different beliefs specifically about the emotion happiness (Joshanloo, 2019). For example, in many non-Western cultures, happiness is commonly believed to lead to bad outcomes, as shown with the Fear of Happiness scale (Joshanloo, 2013). Despite the name of this construct, which at first glance appears to be meta-emotional (i.e., an emotion in response to another emotion; Mendonça, 2013), fear of happiness is defined as a form of “belief.” An example item from this scale: “I believe the more cheerful and happy I am, the more I should expect bad things to occur in my life” (p. 648). As reviewed by Joshanloo (2019), other “conceptions” of happiness that have been studied with specific scales include beliefs about the Inflexibility of Happiness (e.g., “Some people are very happy and some aren’t. People cannot really change how happy they are,” p. 3), Inclusive Happiness (beliefs regarding the relationship between one’s own happiness and that of other entities including friends, country, all living creatures, etc.), Externality of Happiness (e.g., “My happiness is determined by accidental happenings and luck”) and the Fragility of Happiness (e.g., “Something might happen at any time and we could easily lose our happiness,” p. 3). Findings from this line of research provide evidence that these types of happiness beliefs are related to conceptions and experiences of well-being, and further that happiness beliefs found to predominate in one culture do not necessarily generalize to other cultures.

#### 2.2.5 Shame-related beliefs

The experience of shame can be considered to have both beneficial and detrimental effects, especially when considered within a social context. The Shame-Related Beliefs Scale (SRBS) was developed and tested in Chinese samples to assess both aspects (Li et al., 2023). Items in the SRBS refer to one’s own feelings of shame (as opposed to shame in general) and were found to load on one of two factors: enhancing beliefs (e.g., “Sense of shame could make me strive to do better”) and debilitating beliefs (e.g., “Shameful feelings make me doubt my self-worth,” p. 466). Enhancing beliefs about shame were associated with the use of cognitive reappraisal for emotion regulation, and debilitating beliefs with use of an emotion suppression strategy. This tool could be used to study potential cultural differences in self-construal, such as independent self-construal associated with American and many European cultures compared to interdependent self-construal associated with Chinese (studied here) and other Asian cultures.

#### 2.2.6 Stress mindset

One’s stress mindset is described by these authors as the extent to which one does or does not maintain “the belief that stress has enhancing consequences,” including but not limited to improved performance in stressful situations, better health, well-being, growth and learning (Crum et al., 2013). They contrast the “stress-is-enhancing” and “stress-is-debilitating” mindsets. Participants rate their agreement with 8 statements such as “Experiencing stress depletes my health and vitality” and “Experiencing stress enhances my performance and productivity” (p. 732). Six of 8 items refer to one’s self, and thus we consider this scale to primarily relate to beliefs about personal stress as opposed to stress in general. An adaptation of the scale was also developed to assess beliefs associated with a specific stressful situation. The authors found that scores on the Stress Mindset Measure (SMM) predicted physiological reactivity to a stressor, and further that SMM scores could be altered by short educational interventions. This scale was adapted by other researchers to focus on “negative emotion” instead of stress (*Negative emotion appraisals*, described above; Babij et al., 2020).

#### 2.2.7 Theories of anxiety

The Theories of Anxiety (TOA; Schroder et al., 2015) scale was adapted from the ITES, which assesses beliefs about emotions broadly (described above; Tamir et al., 2007), to specifically target malleability beliefs about anxiety (e.g., “You have a certain amount of anxiety and you really cannot do much to change it,” p. 135). Because these items are worded in the second person, we interpret them to target general beliefs about anxiety as opposed to one’s beliefs about their own anxiety. These authors found that TOA and ITES scores were correlated, though relatively weakly (*r* = 0.28), and each uniquely predicted symptoms of mental illness, suggesting that these beliefs are not redundant with each other. A Chinese-language expansion of this scale, which includes depression and stress, has also been developed (*Mindsets of depression, anxiety, and stress*; Zhu et al., 2022).

### 2.3 Beliefs about the emotions of others

In addition to beliefs about emotion in general or about one’s own emotions, researchers have also investigated beliefs held specifically about *other* people’s emotions. For example, Smith et al. (2023) adapted the implicit theories of emotion scale (described above; Tamir et al., 2007) to focus instead on “beliefs about the controllability of another’s emotions.” They found that these beliefs predicted an individual’s willingness to lend interpersonal support to another. Several constructs and scales have focused on the beliefs that adults hold about the emotions of children. For example, Nelson et al. (2012) created a scale to measure parental beliefs about the appropriate (and inappropriate) display of negative emotions by children, and a separate scale to assess beliefs about the social consequences of such displays. The Parents’ Beliefs About Children’s Emotions questionnaire (PBACE) measures beliefs across many dimensions including but not limited to negative consequences, usefulness, controllability of expression, and duration of children’s emotions (Halberstadt et al., 2013). Items from this scale were adapted by Hagan et al. (2020) to create the Teachers’ Everyday Beliefs About Student Anger scale (TBASE – Anger), which taps similar types of beliefs, though focused on beliefs held by teachers, and exclusively for the emotion of anger. Differences in adults’ emotion beliefs depending upon the gender of a child is the focus of the Parents’ Gendered Emotion Beliefs scale (PGEB; Thomassin et al., 2020), which concerns primarily emotion *expression* (e.g., “Children’s personalities, rather than their gender, influence how they expression emotions.”). Not surprisingly, and as demonstrated by all of the researchers cited above, other-focused emotion beliefs hold promise to be especially consequential for the other – the target of the belief.

### 2.4 Additional constructs and measures that overlap with emotion beliefs

It is important and instructive to compare constructs that are exclusively focused on beliefs about emotion, defined and reviewed above, with those that only overlap with this idea. For example, some measures originally developed to assess emotion beliefs are nevertheless confounded with other themes. The Beliefs About Emotions scale was developed to assess people’s beliefs concerning the “acceptability” of experiencing and expressing emotions (Rimes and Chalder, 2010). However, half of the items do not mention emotion, but rather other types of difficult experiences (e.g., “It is a sign of weakness if I have miserable thoughts,” p. 289). It has also been argued that responses on this scale will be sensitive to “an individual’s perception of personal control over emotion,” as opposed to beliefs about one’s emotion *per se* (Veilleux et al., 2015, p. 87). The Rational and Irrational Beliefs about Emotions scales are intended to assess functional and dysfunctional emotion beliefs, respectively (Predatu et al., 2020a). However, a review of individual scale items suggests that this instrument is also likely to be influenced by one’s beliefs about emotional *experiences*. The conflation of beliefs about emotion and about emotion experiences is present within single items, for example “It is very unpleasant to feel negative emotions [*experience*], but I know they are just unpleasant and not something terrible [*belief*]”. Careful delineation between these different classes of statement will be important for the field of emotion beliefs going forward.

Other constructs have been described that intentionally include emotion belief but are not restricted to this construct. For example, the Need for Affect scale was developed to assess people’s tendency and motivations to approach or avoid emotion-inducing situations (Maio and Esses, 2001). An item that exemplifies a tendency is “I feel like I need a good cry every now and then” (p. 591). Other items represent emotion beliefs as defined here, and could be considered as motivations behind why one approaches or avoids emotion, such as “Emotions help people get along in life” (p. 591). The concept of emotion *schemas* also overlaps with, but is not exclusively focused on, emotion beliefs. In the original formulation of this construct, Leahy (2002) pointed out that “individuals differ in their conceptualization and strategies in responding to emotion” (p. 177). As such, although emotion schemas include emotion beliefs (“conceptualizations”), they are broader and specifically include one’s experiences, strategies and perceived self-efficacy in tolerating and regulating emotion (Veilleux et al., 2021a). The breadth of this framework and overlap with other constructs, including emotion beliefs, has likely contributed to some confusion in the literature (Edwards and Wupperman, 2019).

Meta-emotion is a construct that deserves special consideration and comparison to emotion beliefs as defined in the present review. The most widely accepted definition of meta-emotions is *emotions about emotions* (Mendonça, 2013). For example, one may feel angry about feeling sad (Mitmansgruber et al., 2009). However, some authors have argued for a broader interpretation that includes other meta-processes such as “meta-experience of, or meta-cognition about emotion” (Bartsch et al., 2008, p. 11). Here, meta-cognitions about emotion can be considered to include beliefs *about emotions* (e.g., My emotions are meaningful), though may also include beliefs about one’s emotion *attributes*, (e.g., I am able to identify different emotions that I am feeling). Norman and Furnes (2016), taking their lead from the study of meta-cognition, have described a “multifaceted” framework for meta-emotion that includes meta-emotional experiences, strategies, and knowledge. In this view, *meta-emotional knowledge* can be considered to include emotion beliefs, as well as beliefs about one’s tendencies toward certain emotional reactions and response strategies (e.g., When I am angry I find ways to cool down). The concept of *meta-emotional philosophy*, “an organized set of thoughts and metaphors, a philosophy, and an approach to one’s own emotions and one’s children’s emotions” (Gottman et al., 1996, p. 243), also clearly overlaps with, but is not identical to emotion beliefs as defined in the present review. To summarize, many descriptions of meta-emotion overlap with (or even encompass) emotion beliefs. Careful analysis and description of such convergence, as well as divergence, will be important both for the study of meta-emotion and for emotion beliefs. Within the field of meta-emotion research, we recommend that authors take care to point out distinctions between different sub-types of meta-emotional knowledge, including but not limited to beliefs about emotions as opposed to beliefs about one’s own emotional attributes and experiences (see also Norman and Furnes, 2016).

## 3 What’s next?

Many important lines of investigation about emotion beliefs are already being pursued, but the field is still in its infancy and in need of stronger conceptual grounding and careful consideration of methodological approaches, both of which were intended goals of the present review. What is next for this field? Recently, two special issues of scientific journals devoted to emotion beliefs and related constructs have provided wide-ranging suggestions for critical research directions including deepening our understanding of the causes and consequences of emotion beliefs, development of stronger guiding theory beyond simply descriptive frameworks [(e.g., the superordinate vs. subordinate framework of Ford and Gross (2019))], the role of emotion beliefs in the formation and maintenance of psychopathology, other individual differences in specific beliefs, the relationship between emotion beliefs and emotion regulation, and the role of emotion beliefs in cultural norms and maintenance, just to name a few (Gonzalez et al., 2020; Kneeland and Kisley, 2023). As researchers explore these and other areas, we hope they will also consider the importance of different *dimensions* of belief, as described here. How and why might beliefs about different classes of emotion (e.g., positive v. negative emotions; Becerra et al., 2020) differentially impact an individual? Why might one maintain beliefs about emotions in general that are discrepant from their beliefs about their own emotions, and what may be the consequences (e.g., De Castella et al., 2013; Veilleux et al., 2015)? How do beliefs regarding discrete emotions fit together and influence beliefs about emotions more broadly (Gutentag et al., 2023)? How should *emotion* be defined from a layperson perspective? To what extent do laypeople’s belief about emotion correspond to the scientific theories of emotion that are held by researchers? What one “thinks,” “feels,” or “agrees to” may all correspond to belief, but are these all equally strong? How do these propositional attitudes compare to “hunches,” “opinions,” and “knowledge” (e.g., Camina et al., 2021)? Closely related to confidence and degree of conviction is the extent to which beliefs are resistant to change (Connors and Halligan, 2015). Within emotion belief research, there are at least 3 relevant questions to ask here: do emotion beliefs change across different situations and contexts (Ford and Gross, 2019; Veilleux et al., 2021b, 2023); what type and how much contradictory evidence is required before one’s emotion beliefs may change (e.g., Kneeland et al., 2016); and to what extent can emotion beliefs be revised throughout different stages of the lifespan? These and other theoretical questions lead to important methodological considerations as well.

Despite recent advances in developing scales around more clearly defined constructs, and with better divergent validity, there is still room for methodological improvements in the assessment of emotion beliefs. As a simple but potentially impactful example, more careful consideration of scale instructions and item wording can help ensure that instruments are measuring what they are intended to measure. This may include explicitly defining “emotion” for participants, as well as including the words “belief” and “believe” in scale instructions and items. There may also yet be room for improvement in the manner in which the *strength* of one’s belief is assessed. On a Likert-type scale, is endorsing the phrase “I strongly agree” equivalent to endorsing the phrase “I strongly believe?” Because one’s current emotional state has been found to impact the particular emotion beliefs they endorse (Veilleux et al., 2021a, 2023), it may be advisable to assess current affect whenever measuring emotion beliefs. This could allow researchers to account for some variance in emotion beliefs that is not due to the key variable(s) of interest in a given study.

Another important topic that has received too little attention to date is *implicit* emotion beliefs: what are they, what is their relationship to beliefs upon which one can voluntarily reflect, and how do they impact one’s behavior? More and better means of assessing implicit emotion beliefs is also needed. Connors and Halligan (2015) have argued that *most* beliefs are actually implicit, and thus not likely to be consciously reportable through a psychometric scale. They further suggest that when one’s self-report is discrepant with their observed behavior, the latter is more likely to be representative of their actual belief. The investigation of beliefs about emotional residue, described above (Savani et al., 2011), provides a compelling example. Uchida et al. (2009) are among the very few who have investigated implicit emotion beliefs, specifically beliefs about the source of emotions. They were interested in whether people implicitly believe that emotions arise from a “disjointed,” individual self or alternatively from a “conjointed,” collective self. Importantly, they did not directly ask participants to reflect or report on their beliefs. Instead, they reviewed television interviews of Olympic athletes and fans after an event, as well as open-ended descriptions of Olympic athletes’ emotional reactions to an event written by research participants. They rated the extent to which laypeople mentioned other individuals (e.g., family, coaches, friends) when talking about an athlete’s emotions. Such an innovative approach may serve as inspiration for other researchers interested in assessing implicit emotion beliefs.

## 4 Conclusion

Although emotion beliefs have been a recurring topic of interest and research for many decades, it is only recently that the promise of an organized, coherent sub-field of investigation has emerged. However, two interrelated obstacles have been the lack of a clear definition of emotion belief, and a corresponding proliferation of different terminologies, constructs, and measures. As such, many researchers in this area remain unaware of important and relevant work being done by others. The present review aimed to address these obstacles by defining emotion belief, and subsequently re-considering existing constructs and measures that align with this definition regardless of the unique terminologies used by these different researchers. Combined with the understudied research directions and questions highlighted here, we hope this review will help the field of emotion belief continue to expand in ways that will generate informative findings and innovations.

## Author contributions

MK: Conceptualization, Investigation, Project administration, Supervision, Writing – original draft, Writing – review & editing. JS: Conceptualization, Investigation, Writing – review & editing. MM-W: Conceptualization, Investigation, Writing – review & editing. RP: Conceptualization, Investigation, Writing – review & editing.

## Footnotes

^1^For this and the other scales discussed in this paragraph, the quoted phrases are not necessarily provided in the cited publications, but rather are found in the instructions to the actual scales, which were shared with us by the respective authors.

^2^This is not the same definition of *attitude* commonly employed in social psychology, which implies an evaluative or preferential mental state (Harmon-Jones et al., 2011; Connors and Halligan, 2015).

^3^For further discussion of this issue and an alternative viewpoint to that expressed here see Camina et al. (2021).

^4^For this we reviewed the following dictionaries: Merriam-Webster, Dictionary.com, Cambridge Dictionary, and Collins Dictionary.

^5^This wording choice was deliberate. The authors choose to avoid the phrase “negative emotion” because many laypeople may not be familiar. Further, some sub-populations of participants with psychopathology consider happiness and other affective states described in scientific literature as “positive” to be, in fact, negative (Rachel Manser, personal communication).
